# Improvements for better scaling of locally managed marine areas

**DOI:** 10.1111/cobi.70091

**Published:** 2025-06-19

**Authors:** Emily Lewis‐Brown, Hope Beatty, Katrina Davis, Ando Rabearisoa, Jeannot Ramiaramanana, Robert M. Ewers, Morena Mills

**Affiliations:** ^1^ Science and Solutions for a Changing Planet DTP, Department of Life Sciences Imperial College London Berks UK; ^2^ Department of Life Sciences Imperial College London Berks UK; ^3^ Department of Biology University of Oxford Oxford UK; ^4^ Conservation International Madagascar Antananarivo Madagascar; ^5^ Conservation Action Lab UC Santa Cruz Santa Cruz California USA; ^6^ Department of Ecology & Evolutionary Biology University of California, Santa Cruz Santa Cruz California USA; ^7^ Department of Economy University of Antananarivo Antananarivo Madagascar; ^8^ Georgina Mace Centre for the Living Planet Imperial College London Ascot UK; ^9^ Centre for Environmental Policy Imperial College London London UK

**Keywords:** diffusion of innovation theory, IPA, LMMAs, marine protected areas, NPS, sustainable fisheries, AIR, AMML, áreas marinas protegidas, pesquerías sustentables, PNP, teoría de difusión de la innovación, 当地管理的海洋区域(LMMAs), 海洋保护区, 可持续渔业, 创新扩散理论, 重要性‐绩效分析(IPA), 净推荐值(NPS)

## Abstract

To protect and restore ecosystems at the speed and scale required to meet current environmental challenges, a greater understanding of how conservation initiatives spread from existing to new adopters is required. According to the diffusion of innovation theory, positive adopter‐to‐peer communication is a powerful driver of innovation spread, whereas negative communications hinder innovation spread. Aware of this, businesses regularly survey customers and respond accordingly to maximize company growth. Therefore, we used 2 consumer satisfaction research measures commonly used by businesses, importance–performance analysis (IPA), which measures performance on metrics that are most important to customers, and net promoter score (NPS), which measures likely spread through positive referrals, to study satisfaction among adopters of locally managed marine areas (LMMAs) in northeastern Madagascar. Our results identified 4 attributes of LMMAs that adopters viewed as important but rated as worsening over time (funding and livestock provided by a nongovernmental organization, conflict in the village, and connections with others). Adopters considered control over resources and fisheries restrictions important and high performing. Villagers rated their quality of life since adopting LMMAs positively on average, but NPS returned a negative result overall and a strongly negative score for nonleaders. Our findings can be used to improve the design and management of LMMAs, inform pre‐ and postproject impact assessments to minimize negative impacts from conservation initiatives, and increase the spread of conservation initiatives. More broadly, this study presents a novel outlook for increasing the adoption of conservation initiatives by framing adopters of conservation initiatives as akin to customers whose perceptions of conservation initiatives matter inherently and because of their power to influence the spread of conservation initiatives.

## Abstract (es)

Mejoras en la escala de las áreas marinas con manejo local

**Resumen:**

Para proteger y restaurar los ecosistemas a la velocidad y escala necesarias para enfrentar los retos ambientales actuales, es preciso comprender mejor cómo se difunden las iniciativas de conservación de los adoptantes existentes a los nuevos. Según la teoría de difusión de la innovación, la comunicación positiva entre adoptantes es un impulsor potente de la difusión de la innovación, mientras que las comunicaciones negativas la obstaculizan. Conscientes de ello, las empresas encuestan periódicamente a sus clientes y responden en consecuencia para maximizar el crecimiento de la empresa. Para estudiar la satisfacción de los usuarios de las áreas marinas con manejo local (AMML) en el noreste de Madagascar, utilizamos dos medidas de satisfacción del consumidor utilizadas habitualmente por las empresas: el análisis importancia‐rendimiento (AIR), que mide el rendimiento en función de las métricas más importantes para los clientes, y la puntuación neta del promotor (PNP), que mide la posible difusión a través de referencias positivas. Nuestros resultados identificaron cuatro atributos de las AMML importantes para los adoptantes, pero que consideraban empeoraban con el tiempo (financiación y ganado proporcionados por una organización no gubernamental, conflicto en el pueblo y conexiones con otros). Los adoptantes consideraron importante y de alto rendimiento el control sobre los recursos y las restricciones pesqueras. En promedio, los aldeanos valoraron positivamente su calidad de vida desde la adopción de las AMML, pero el PNP arrojó un resultado negativo en general y una puntuación muy negativa para los no líderes. Nuestros hallazgos pueden utilizarse para mejorar el diseño y la gestión de las AMML, informar las evaluaciones de impacto previas y posteriores a los proyectos para minimizar los impactos negativos de las iniciativas de conservación y aumentar la difusión de las iniciativas de conservación. En términos más generales, este estudio presenta una perspectiva novedosa para aumentar la adopción de iniciativas de conservación, al enmarcar a los adoptantes de iniciativas de conservación como algo similar a clientes cuyas percepciones de las iniciativas de conservación importan intrínsecamente y debido a su poder para influir en la difusión de las iniciativas de conservación.

## 扩大当地管理的海洋保护区域规模的改进措施

**【摘要】** 应对当前环境挑战, 需要以一定的速度和规模来保护并恢复生态系统, 这依赖于深入理解保护措施如何从现有的保护行动接受者向新接受者扩散。根据创新扩散理论, 接受者与同伴间的积极沟通是创新扩散的强大驱动力, 而消极沟通则会阻碍创新扩散。意识到这一点的企业会定期调查客户并做出相应调整, 以实现公司增长最大化。因此, 本研究采用了企业常用的两种客户满意度研究指标:用于衡量客户最重视指标的绩效表现的“重要性‐绩效分析”, 以及用于衡量通过积极推荐实现扩散可能性的“净推荐值”, 来探究马达加斯加东北部当地管理的海洋区域(locally managed marine areas, LMMAs)保护行动接受者的满意度。研究结果识别出接受者认为重要但随时间推移逐渐恶化的四个LMMAs属性(非政府组织提供的资金以及牲畜、村庄内部冲突、与他人的联系)。接受认为对资源的控制和渔业限制措施均十分重要且效果良好。村民对建立LMMAs后生活质量的平均评价为积极评价, 但净推荐值整体结果较为负面, 且非领导者群体给出了强烈的负面反馈。我们的研究成果可用于改进LMMAs的设计与管理, 为项目实施前后的影响评估提供依据, 以减少保护措施的负面影响, 并促进保护措施的推广扩散。更广泛而言, 本研究提出了一种新的视角, 即通过将保护措施的接受者视为类似于客户的群体, 承认其对保护措施的认知既具有内在重要性, 且其影响保护措施推广的能力至关重要, 以促进保护措施的扩散。**【翻译:胡怡思;审校:聂永刚】**

## INTRODUCTION

Coasts and oceans provide food, fuel, and fiber for billions of people and support social and cultural well‐being (Alongi, 2002; Marshall et al., 2019). These rich and diverse environments are under unprecedented pressure and require greater adoption and spread of effective conservation initiatives within a decade (Laffoley et al., 2020; Purvis et al., 2019; UNEP, 2019). The adoption of marine conservation initiatives is motivated by the balance between anticipated benefits and costs, especially those aligning with adopters’ values (Harvey et al., 2018; Mills et al., 2019). However, understanding what helps or hinders the spread of conservation initiatives within or between communities remains limited but is critical for ensuring enduring impact (Mascia & Mills, 2018; Romero de Diego et al., 2021).

**FIGURE 1:**
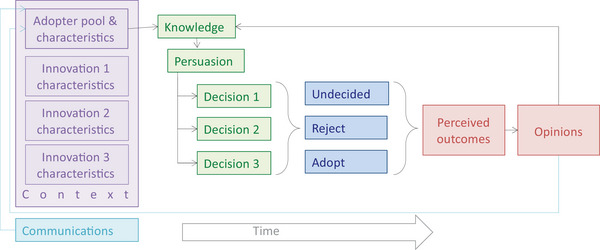
Diffusion (spread, adoption, and coadoption) of conservation innovations based on Rogers (2003), Dearing (2008), and Wisdom et al. (2014) (purple, characteristics of innovations, adopters, and their context; green, decisions; blue, adoption or not of one or more innovations; red, outcomes and consequent opinions over time; light blue, adopters’ opinions affect knowledge of existing and potential adopters and external sources communicate additional knowledge to potential adopters).

Processes and factors involved in the adoption and spread of innovations are described in the diffusion of innovation theory (hereafter, diffusion theory) (Rogers, 2003). This theory informs the purposive diffusion of technologies and behaviors in a range of fields (Dearing & Cox, 2018) and can help catalyze conservation at larger scales (Mascia & Mills, 2018). Adoption flows from knowledge (e.g., through information from existing adopters, governments, or charities) through persuasion to decisions to reject or adopt innovations, which lead to outcomes (Figure 1). Outcomes are often assumed to be positive due to pro‐innovation bias (Rogers, 2003), but negative outcomes are possible, and outcomes result in adopters forming positive or negative perceptions and consequent opinions (Rosenberg, 2001). If communicated, these opinions can influence potential new adopters and either increase or limit the spread of innovations (Dearing & Cox, 2018). Influence is not uniformly distributed among individuals in a social network (Mbaru & Barnes, 2017) and is strongest between near peers, especially with negative opinions (Dearing & Cox, 2018; Ramirez et al., 2014). Avoiding negative opinions and communications is critical in influencing the spread of innovations (Dearing, 2009) and also important for ethical reasons (Woodhouse et al., 2015). Aware of this, businesses monitor customer satisfaction and opinions because they correlate with consumer behavior and company success and growth (Azzopardi & Nash, 2013; Blasberg et al., 2008; Sun & Kim, 2013).

Research evaluating conservation initiatives has focused on measuring ecological and some social outcomes (Edgar et al., 2014; Jupiter et al., 2017; Oldekop et al., 2016), but satisfaction among those engaged in conservation is rarely measured and remains poorly understood. Conservation initiatives, for example, marine protected areas (MPAs), routinely report outcomes, such as increased fish abundance and biomass within MPAs (Garcia‐Rubies et al., 2013; Gilchrist et al., 2020) and surrounding seas (Kough et al., 2019). These outcomes can increase local people's fishery catches and wealth (Long, 2017; Robertson et al., 2020) but are site specific and require case‐by‐case evaluation (Hilborn et al., 2004).

Although these objective metrics may contribute to understanding changes in various aspects of human well‐being, people's subjective opinions of their satisfaction, relationships, and quality of life are also important for people affected and for the spread of conservation initiatives (Loveridge et al., 2020; Mbaru et al., 2021). Monitoring and evaluation should include metrics that matter most to those affected (Woodhouse et al., 2016), and by understanding the meaning of conservation initiatives to their adopters, initiatives can be tailored to increase the chances that they spread through positive referrals.

Reported negative outcomes and impacts of MPAs include conflict and displacement of local communities, raising ethical concerns (Bennett et al., 2017; de Lange et al., 2016; Oldekop et al., 2016) and potentially reducing the success and spread of conservation initiatives. To minimize negative impacts on resource actors, conservation is shifting from top‐down towards community‐based initiatives (Spalding et al., 2016) and comanaged conservation (Oldekop et al., 2016). Additionally, governmental, nongovernmental, and academic authors recommend voluntary measures to predict and minimize negative impacts of conservation on local communities for ethical reasons (Bennett et al., 2017; CIHR, 2010; Greiber, 2009), including respect for Indigenous peoples as rights holders (Hoyle, 2011; UNFAO, 2016). However, voluntary measures are considered “not appropriate in situations where high rates of participation and compliance are required, where there is limited flexibility regarding actions and timings, or where serious social or environmental risks are involved” (RSPB, 2015). To minimize and mitigate negative impacts from certain large‐scale projects, industrial developers in many countries are legally required to predict, minimize, and compensate for negative impacts on people and their environment, with monitoring prior to, during, and after development (EU, 2011; Saeed et al., 2012; Wathern, 1992). Similarly, research institutes require ex ante impact assessments and ethical evaluations and monitoring, with compensation for negative impacts on those undertaking or affected by research (Brittain et al., 2020).

Although evaluations at all stages of a project are intrinsically linked, we focused on the subjective satisfaction levels and opinions of people who previously adopted and remain engaged with conservation initiatives. For this, we studied adopters’ satisfaction with locally managed marine areas (LMMAs) in 4 villages in northeast Madagascar, each of which had established an LMMA 10 years previously. In doing so, we recognize adopters of LMMAs as the rights holders, resource users, and custodians of their environment, and conceptualize them as akin to customers whose opinions matter inherently and for the success and spread of conservation initiatives.

LMMAs and MPAs include area‐based initiatives that conserve fish populations by restricting harvesting partially or totally (Spalding et al., 2016). MPAs are categorized into different types by the International Union for Conservation of Nature (IUCN); they must prioritize nature conservation; and they must be designated by law or an authoritative rule maker (Grorud‐Colvert et al., 2021). LMMAs, and other effective conservation measures (OECMs), are not MPAs and do not need to prioritize nature, but they may provide some conservation benefits (Grorud‐Colvert et al., 2021). Although MPAs may be established by communities, they are commonly established by governments or international bodies. LMMAs, however, are locally designed and managed and are often comanaged between communities, nongovernmental organizations (NGOs) or government agencies, or both, and some have legal designation (Newell et al., 2019; Robertson et al., 2020). Several studies report positive outcomes from LMMAs, and LMMAs in Madagascar are spreading and enduring (Gardner et al., 2020; Harris et al., 2012; Long et al., 2019; Newell et al., 2019; Oliver et al., 2015; Rocliffe et al., 2014). We were interested in determining whether satisfaction by local communities reflected this.

We used importance–performance analysis (IPA) (Martilla & James, 1977), which identifies and prioritizes which factors of a product or service need targeting to improve adopter satisfaction (Sever, 2015). IPA is adaptable to a range of settings (Azzopardi & Nash, 2013) and is used widely in health care, education, and tourism (Abalo et al., 2007; Sever, 2015). However, IPA has been used only once in conservation evaluation—in a study of adopters’ satisfaction with community management areas in Ghana (Agyare et al., 2015). Importance and performance are subjective concepts, and we sought to consider adopters’ personal perspectives.

We also calculated the net promoter score (NPS), which indicates customer satisfaction and can be used to predict the growth or spread of a product, service, or company (Borsci et al., 2020; Laitinen, 2018; Reichheld, 2003. Its use is relatively new in conservation evaluation. NPS can also be used to identify groups or individuals in communities who are dissatisfied; addressing their concerns is key to turning adopters into promoters (Blasberg et al., 2008). We provide devised recommendations for how it might be possible to measure and improve adopter experiences and perceptions of a conservation initiative that may have relevance more broadly.

## METHODS

### LMMAs and the study site

LMMAs are community‐based initiatives to manage marine resources and wildlife in a specified area (Govan, 2009). With a long and broad history across the Pacific islands, LMMAs are based on local traditional customary tenure (Jupiter et al., 2014), and their establishment is often supported with financial and logistical help from NGOs (Rocliffe et al., 2014). LMMAs form around locally agreed rules to manage and restore coastal fisheries, habitats, and wildlife, and encompass various management measures, such as prohibiting some fishing gears, such as small‐holed nets, protecting corals from physical damage, protecting and planting mangrove trees, and temporary or permanent closures (Westerman & Benbow, 2013). Impacts of periodic closures in LMMAs include improved fishery harvests and incomes (Benbow et al., 2014; Gardner et al., 2020; Gilchrist et al., 2020). For example, closures for 3–6 months in southwestern Madagascar increased incomes because octopus double in body weight monthly and large octopus are worth more per kilo than smaller animals (Mayol, 2013).

We studied satisfaction with the outcomes of LMMAs in 4 villages on the northeast coast of Madagascar, in the regions of Mahavanona and Ramena. The establishment of LMMAs in this biodiverse yet vulnerable region has been encouraged by the Malagasy government and NGOs (Zorzi & Rabearisoa, 2013). An NGO initiated an MPA in the area in 2009, one of the first in Madagascar, but it was initially resisted by local villages (MIHARI, 2015). However, the NGO collaborated with the fisheries association in each village that established LMMAs between 2009 and 2013. In 2015, the Ambodivahibe MPA was formally established by decree 753 in the waters adjacent to the LMMAs of the 4 villages studied. The area is considered important for conservation because of its natural resilience to climate change due to cool water upwelling in 2 deep bays in the MPA, but is at risk from reduced rainfall and damaging fishing practices (Belokurov et al., 2016). The NGO provided training, facilitation, funding, and livestock. The villages and NGO are not named for anonymity. The villages studied mainly rely on subsistence fishing with some livestock raising and are each home to 100–300 residents, plus seasonal migrant fisherfolk.

Once designed, ethics permission for the study was provided by Imperial College London (2018‐01438539‐BEATTY‐HB). Consent for the research was then secured from the Malagasy government through the University of Antananarivo and from village leaders prior to conducting key informant interviews, focus groups, and in‐person surveys. Key informant interviewees mentioned that the villages had fisheries associations prior to the establishment of LMMAs and measures to manage their fisheries. The management measures undertaken in the LMMAs varied and included temporary closures for prawn or lobster harvesting and permanent no‐take areas of all species in important fish‐feeding areas. Some LMMAs banned small‐holed nets, ring nets, spear guns, and damage to mangroves and corals. Some villages already had dinas, charters of community‐based customary rules sometimes recognized in Malagasy law (Klein, 2024). Interviewees mentioned the NGO involved had helped villages formalize their dinas and had contracts with some of the study villages about their fisheries management, although details were not provided.

### Survey instruments and surveys

We developed a survey instrument (Appendix S1) to test respondents’ attitudes toward and satisfaction with LMMA outcomes across a list of attributes. Attributes that influence the adoption of innovations (Cetas & Yasue, 2017; Farmer et al., 2011; Rogers, 2003) and those typically used to evaluate marine conservation outcomes (Ahmadia et al., 2015; Ban et al., 2019; Bennett & Dearden, 2014; Mascia et al., 2017) were included. An initial list of 35 attributes was reduced and reworded to be locally relevant through feedback from 4 focus groups held by the research team in villages close to the survey sites in Madagascar in the local dialect of Antakarana. The survey had 3 sets of questions: best–worst scaling (BWS) choice experiment to rank the importance of LMMA attributes; scale‐based questions to rate the performance of LMMA attributes; and questions about adopters’ age, gender, income, role in the community (leader or nonleader), livelihood, education, and opinions of LMMAs (Appendix S1).

First, local researchers met with village leaders, who gave information and permission for surveys and indicated fisherfolk who might complete the survey and the fishery landing areas where fisherfolk may be found. Respondents were identified to include individuals representing a range of opinions and heterogeneity (those self‐reporting in the survey as males, females, village leaders, and nonleaders) (Table 1). This combined purposive and snowball sampling (Robinson, 2013) has been used elsewhere with BWS (Davis et al., 2015) and IPA (Agyare et al., 2015). Survey responses were subject to unavoidable memory errors (Kamper et al., 2009). Due to nonrandomized sampling and memory bias, analyses were limited to descriptive rather than inferential analyses, and reporting of results is limited to the sample only. We made no wider generalizations.

**TABLE 1:** Distribution of samples of responses to a survey on attitudes toward and satisfaction with locally managed marine area outcomes (*n* = 88).

	Female	Male	Female	Male	
Survey location	Leader	Leader	Nonleader	Nonleader	Total
Village 1	5	11	1	3	20
Village 2	1	8	5	9	23
Village 3	2	7	5	8	22
Village 4	3	9	7	4	23
Total	11	35	18	24	88

Local enumerators gained prior, informed consent from respondents to participate in the study. Consent was audio‐recorded or provided in writing. These enumerators surveyed 110 residents across 4 villages in May–June of 2018. All respondents lived in the villages surveyed, and most respondents (98%, *n* = 84) were involved in the decision to establish their village's LMMA. Responses of respondents not present during the LMMAs’ establishment were excluded from analyses, as were those who failed to complete >60% of both best and worst responses in the choice experiment. These restrictions left us with 88 usable surveys. Data were translated into English, and answers to open‐ended questions were coded inductively based on keywords and themes.

### Importance–performance analyses

IPA surveys typically invite respondents to rate the importance of a number of the attributes of an innovation or service with scale‐based questions (Azzopardi & Nash, 2013; Lai & Hitchcock, 2015; Martilla & James, 1977). Respondents then rate the performance of the innovation or service on the same scale and list attributes to give 2 sets of scale‐based data. However, variations on this method are possible (Abalo et al., 2007). We plotted the average (mean) importance and performance scores for each attribute on an IPA matrix plot divided into 4 quadrants (Azzopardi & Nash, 2013; Lai & Hitchcock, 2015; Martilla & James, 1977).

**FIGURE 2:**
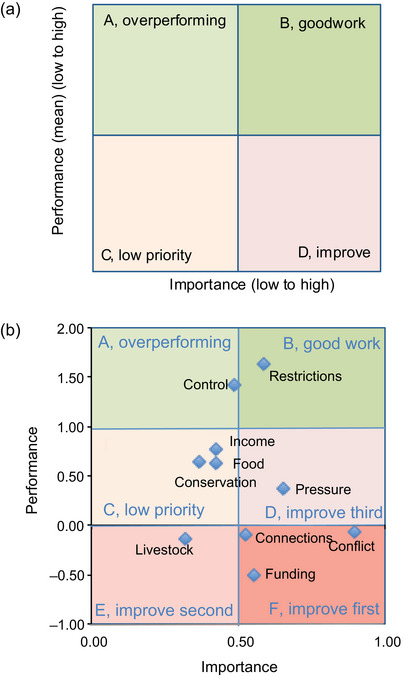
Importance–performance matrices: (a) a simple matrix with 4 scale‐centered quadrants and (b) the average importance results relative to average performance results from surveys of 88 adopters of locally managed marine areas (LMMAs) in northeast Madagascar for 10 attributes of LMMAs (*n* = 88) (A, savings may be possible because performance is high on unimportant attributes; B, important and high‐performing attributes; C, low‐priority attributes because they are unimportant to adopters; D, attributes require improvement because they are highly important but perform poorly; negative performance values, attributes that worsened since LMMA establishment [E and F]). Performance was measured through scale‐based questions with response options from *much better* (+2) to *much worse* (−2, truncated to −1 to fit observations). Importance (*x*‐axis) is measured through a choice experiment (best plus worst).

We categorized the quadrants of the matrix plot to indicate where action should be prioritized to optimize the balance between respondent satisfaction and resource investment (Figure 2a). Quadrant A indicated attributes for which performance was greater than importance, labeled *overperformance* (i.e., savings could be made). Quadrant B contained attributes for which the innovation or service performance was similar to importance and both were high, labeled *good*. Quadrant C indicated attributes for which an innovation could be performing better but importance to adopters was low, labeled *low priority*. Attributes rated as high in importance but poor in performance were in quadrant D and were the priorities to improve, labeled *improve*. Whether importance and performance ratings were plotted on the *x*‐axis or *y*‐axis is a matter of preference, and the quadrants are adjusted accordingly. The threshold lines between quadrants of the IPA plot can be set in different ways: average (mean) of the results for data‐centered plots; central points of rating scales for scale‐centered plots; and performance targets (e.g., targets for outcomes predetermined by the organization commissioning the research) (Azzopardi & Nash, 2013; Tonge & Moore, 2007).

To measure importance, we asked respondents to rank each attribute (e.g., first, second, third) rather than rate them (e.g., good, fair, poor), a recommended refinement that provides a better measure of importance (Abalo et al., 2007). Respondents ranked attributes of LMMAs using a case 1 BWS choice experiment, providing a quantitative relative ratings scale of attributes (Finn & Louvier, 1992). Each survey presented 8 choice sets, each with 4 LMMA attributes, from which respondents indicated the best and worst attributes. A total of 16 attributes were assessed, and respondents saw each attribute twice. Eight survey versions were used to balance attribute coappearance. A score of 1 was allocated to each attribute when it was selected as either the best or worst. A score of zero was assigned if an attribute was not selected. We calculated the number of times attributes were selected as a proportion of their occurrence instances in the choice experiment. This provides relative ranked data on a scale of 0–1, which we scaled up (multiplied by 2) to occupy the same range as performance values, after Abalo et al. (2007). First, we calculated importance as best minus worst, then as best plus worst. The 2 methods provide similar results, but we report best plus worst to simplify the IPA plot.

**FIGURE 3:**
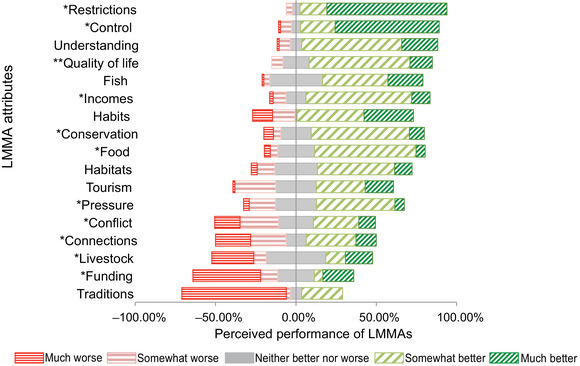
Performance rating from much worse (−100) to much better (100) of attributes of locally managed marine areas (LMMAs) in northeast Madagascar based on surveys of 88 adopters of LMMAs, showing the percentage of responses for each question per category (*n* = 88) (*, attribute used in the IPA; **, attribute used for the net promoter score in Figure 4).

To measure performance, each respondent was presented with 5‐point scale‐based questions, anchored with descriptive adjectives (Harpe, 2015). Options ranged from *much better* to *much worse* after the global rating of change scales (GRCS) (Kamper et al., 2009). The scale included 5 approximately evenly spaced categories (coded in the analysis from −2 to +2) around a neutral center of *neither better nor worse* (0) (Harpe, 2015). The central scores, therefore, indicated that if there was a change, it was neither better nor worse, rather than no change occurred. We plotted the mean, median, and mode of the performance scores (Appendix S2.1), and these measures were the same in many cases. It is standard to report the mean (average) in research using IPA and GRCS to measure perceptions (Azzopardi & Nash, 2013; Fischer et al., 1999; Kamper et al., 2009). However, using means of data from scale‐based questions can be controversial (Sullivan & Artino, 2013) because scale‐based categories may not be equally sized and spaced and means may not be meaningful (Robbins & Heiberger, 2011). However, the numbers attributed to the categories are reported to be independent of these characteristics and therefore may be used to make inferences about their means (Harpe, 2015; Norman, 2010). We used mean preference scores to describe the average central value of adopters’ perceived relative performance of attributes. Presenting averages of data may mask polarized views (Sullivan & Artino, 2013); therefore, we present the distribution of all performance ratings (Figure 3; Appendix S2.1) and use nonparametric tests to examine the data further (detailed below).

We plotted results for 10 attributes with both importance and performance data on a scale‐centered IPA plot and extended the IPA matrix to represent ratings of much worse as negative values, adding zones E and F. Following standard IPA nomenclature, we made the following categorizations: E, improve second; F, improve first (Figure 2b). We used standard scale‐centered thresholds because it was logical to place the quadrant threshold at zero in the performance scale (a 5‐point scale from *much worse* [−2], to *neither better nor worse* [0], to *much better* [+2]). Further details on undertaking IPA are in Appendix S2.

### Opinion analyses

We conducted a principal component analysis (PCA) to identify attributes with similar response patterns (Ryan & Sterling, 2001). We limited the PCA to the 10 attributes used in the IPA for consistency and to accommodate the small sample size (Reise et al., 2000). To determine the full spread of opinions captured in the survey, we analyzed all 18 performance questions with descriptive analyses (Appendix S1, questions 24–41). We used Pearson chi‐square to test for the association between self‐reported gender and leadership roles. We used Fisher's exact test to explore differences between genders and roles and how they rated their level of involvement in the adoption decision, and LMMA performance. We used Spearman's Rho to test for correlations between the level of involvement and performance ratings.

To complement the IPA, which we used to identify specific attributes for improvement to increase overall satisfaction most, we also calculated the NPS, which indicates satisfaction among differing sections of the customer community so that their opinions can be addressed to increase the likelihood of spread (Blasberg et al., 2008). As with previous studies, we deviated from the standard NPS question (Morgan & Rego, 2006) and used the performance question: Has the overall quality of life changed in the village? We used this question because it is recommended that subjective quality of life be surveyed (Woodhouse et al., 2016). NPS typically has an 11‐point response scale (0–10). Those responding 10 and 9 were considered promoters, those responding 8 and 7 were considered passive, and those responding 6 or below were considered detractors. Because our survey instrument was not delivered visually, to accommodate low literacy rates, we contracted the scale and used a 5‐point scale. In line with NPS, we scored responses of 5 as promoters, 4 as passive, and 3 or below as detractors and calculated the NPS as the number of promoters minus the number of detractors (Laitinen, 2018). We provide templates for the IPA and NPS online under the Creative Commons Attribution License CC BY‐NC (Lewis‐Brown, 2024).

Finally, to gain qualitative insights into respondents’ opinions, we undertook content analysis of open‐ended questions in the survey (Appendix S1). Responses were coded and categorized into themes with minimal alteration of terms to remain as faithful as possible to respondents’ sentiments (Graneheim et al., 2017).

## RESULTS

The results indicated more dissatisfaction than satisfaction with the LMMAs. Five attributes were said to require improvement, 3 were ranked as low priority, and 2 were perceived as performing well, and the NPS was negative (−9.31). Despite this, most respondents (98%, *n* = 86) wished to keep their LMMAs.

### Importance, performance, and their relationship

Two attributes were rated as important and performing well (restrictions on fishing, and control over the resource) (Figure 2b). Four attributes were perceived to have worsened since the LMMAs were established (Figure 2), 2 of which were particularly notable. These included the most important attribute: conflict within and between villages. Conflict was selected almost every time it appeared in choice tables (always as worst), indicating its importance to respondents, and received a negative performance score (mean −0.90). This result placed conflict in zone F of the IPA plot (Figure 2b) and prioritized conflict as the first priority for improvement. Respondents also perceived funding and training provided to villages adopting LMMAs as important but performing poorly; 53% of respondents rated this attribute negatively (*n* = 59). Open‐ended question responses were vague about the nature and causes of conflicts from the LMMA, and this issue was raised in response to different survey questions. One respondent cited conflict as a reason for not wanting the continuation of the LMMA. Another cited unfair sharing of benefits, which was a recurring response to several open‐ended questions. One respondent linked conflict to changes in incomes since establishing their LMMA, but further detail was not given. Conflict was attributed to weakening of traditions by one respondent.

Conversely, restrictions of certain activities in an LMMA (e.g., temporary closures and gear restrictions) were the only attribute plotted in the good work zone B. This attribute was the third most important and best‐performing attribute of LMMAs. Control over the resources was also scored as performing well, and of some importance to adopters.

### Opinions of LMMA outcomes, abandonment, and spread

The PCA identified patterns in the scale‐based performance ratings and showed 4 clusters of attributes with similar performance ratings patterns (Appendix S2.8). The primary component (C1, nature conservation, pressure on the resource, and food) was coded *natural resources*. The second component (C2, livestock and funding provided to villages establishing LMMAs) was coded *NGO support*. The third component (C3, fishing restrictions, incomes, and control over decisions) was coded *self‐determination*, and C4 (conflict and connections with other people) was coded *relationships*. Considering the PCA (Appendix S2.8) and IPA results (Table 2), attributes in C2 (NGO support) and C4 (relationships) required the greatest improvement because they were important to respondents but received negative mean performance ratings. In contrast, the primary components C1 (natural resources) and C3 (self‐determination) received more positive ratings. Therefore, LMMAs appeared to be rated by adopters as performing better on attributes relating to natural resources and self‐determination than on NGO support and relationships.

**TABLE 2:** Results of surveys on the importance and performance of 10 attributes of locally managed marine areas (LMMAs) established by local communities, often with support from government or nongovernmental organizations.^a^

Attribute	Abbreviation (PCA component)^b^	Importance	Performance
Functioning of the environment (nontarget species)	Conservation (C1)	0.74	0.64
Food we could catch to eat	Food (C1)	0.85	0.63
Pressure on the resources due to more people fishing	Pressure (C1)	1.31	0.37
Incentives for villages to establish and run an LMMA	Livestock (C2)	0.64	−0.14
Funding and training provided by an NGO	Funding (C2)	1.1	−0.51
Restriction of certain activities in an LMMA	Restrictions (C3)	1.17	1.63
Control we would have over our marine resources	Control (C3)	0.98	1.42
Amount of money we could earn	Income (C3)	0.85	0.76
Connections to people beyond the village	Connections (C4)	1.05	−0.09
Level of conflict in or beyond the village	Conflict (C4)	1.8	−0.07

Abbreviations: LMMA, locally managed marine area; NGO, nongovernmental organization.

^a^ The range and frequency of performance responses are in Figure 3 and Appendix S2.1. Importance data are weighted (doubled) to the same scale as performance.

^b^ Principal component analysis (PCA) components are in parentheses and rated based on performance data.

In total, respondents rated the performance of LMMAs on 18 attributes, 13 of which were rated positively and 5 negatively based on mean rating scores on the scale from −2 (much worse) to +2 (much better) (Figure 3), and the mean of means was positive (0.51). The most negatively performing attribute was changes to village traditions and customs since establishing the LMMA; 65% of respondents rated traditions as much weaker since establishing LMMAs (mode = −2, median = −2, mean = −1.07, *n* = 83). Although these data did not show causation, open‐ended questions offered insights. For example, one respondent said,

I don't want the continuation of the LMMA because it has changed many things in the village. It has created lots of conflicts…. I don't know why there is this disrespect of our traditions.

Another respondent linked changes in conflicts to changes in traditions, and several respondents mentioned supporting the traditional dina rules to manage their seas. Likewise, funding was rated negatively, along with livestock, connections to others, and conflict. The remaining 13 attributes were rated positively on average, particularly the restrictions on fishing and control over resources.

When performance ratings from leader and nonleader respondents were compared, large areas of consensus appeared. However, leaders rated LMMA performance significantly more positively than did nonleaders on 2 attributes: conflict (Fisher's exact test = 16.59, *p* = 0.002, *n* = 88) and traditions (Fisher's exact test = 10.03, *p* = 0.008, *n* = 83) (Appendix S2.4). Leaders also reported being more involved in the decision of whether to adopt the LMMA than nonleaders did (Fisher's exact test = 11.21, *p* = 0.016, *n* = 85). Involvement in the adoption decision was not significantly correlated with 14 of the 18 LMMA attributes. Significant negative correlations were found between involvement in the adoption decision and 4 attributes. It correlated negatively with connections (coefficient −0.274, *p* = 0.012, *n* = 84), traditions (coefficient −0.373, *p* = 0.001, *n* = 82), and funding (coefficient −0.285, *p* = 0.032, *n* = 57) and correlated positively with fisheries restrictions (coefficient 0.277, *p* = 0.013, *n* = 80). However, the coefficients were not high, and causation was not tested or implied.

**FIGURE 4:**
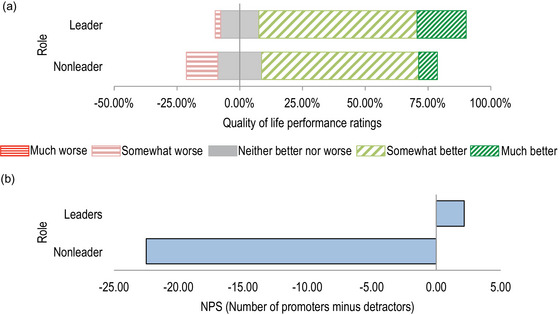
Rating of changes in the quality of life of respondents since adopting locally managed marine areas (LMMAs) as per leaders (*n* = 40) and nonleaders (*n* = 46) in northeast Madagascar who answered this question: (a) performance ratings on a 5‐point Likert‐style scale from *much worse* to *neither better nor worse* to *much better* (leaders, village representative, council member, president, or vice president mean = 1.00; nonleaders mean = 0.65) (The range of categories is from somewhat worse to much better because no respondents said their quality of life was much worse since adoption of the LMMA.) and (b) net promoter score (NPS) for leaders (2.17) and nonleaders (−22.5).

When the same tests were run for self‐reported gender groups, no significant differences were found. Considering adopters’ perceptions of changes in their quality of life since establishing LMMAs (*n* = 86), the mean performance rating (from −2 to +2) was positive (mean = 0.84), with no significant difference between village leaders and nonleaders (Fisher's exact test = 5.3, *p* = 0.143) (Figure 4a). However, the NPS (Figure 4b) result was negative (NPS −9.31) because this metric discards somewhat better responses as passive (62.79% of responses) and calculates the percentage of promoters (13.95%) minus detractors (23.26%). The NPS differed between village leaders, who were primarily promoters (NPS +2, *n* = 46), and nonleaders, who were primarily detractors (NPS −22, *n* = 40). Finally, the NPS data (coded as +1 for promoters, 0 for passives, and −1 for detractors) differed significantly from the performance ratings data (coded as +1 for better ratings, 0 for neither better nor worse, and −1 for worse ratings) (Fisher's exact test value 80.8, *p* ≤ 0.001). This was because the majority of respondents rated performance as somewhat better (62.79%), which scored 0 (passive) in the NPS but scored +1 when calculating simple average data.

When asked whether respondents would like to keep their LMMA, almost all respondents (98%) reported they wished to keep their LMMA. When asked to explain why, 4 main reasons were mentioned (Appendix S7): “to protect the sea and resources” (41%); “increase catches” (15%); “for current benefits” (16%), and “for future generations” (10%). For example, one respondent commented: “With no LMMA the people are poor, but with the LMMA, the people are no longer in poverty” and “You can see lots of benefits and it develops your way of living.” However, 18% of respondents expressed discontent with the LMMA management. Nine respondents (including leaders and fisherfolk) cited unequal distribution of livestock incentives as problematic, stating that everyone in the village should benefit, not just fisherfolk. Two respondents (from Village 2) did not wish to keep their LMMA, and one explained that “When they share something, some of us don't benefit.” Some respondents volunteered that they wished for other villages to join their LMMA, and one wished for other villages to establish LMMAs. Several respondents mentioned that LMMAs had spread between villages through exchange visits and peer communication. For example, one respondent in Village 2 commented: “We have seen the success of [another village with an LMMA], so that convinced us.”

When asked to compare MPAs to LMMAs, most of those who commented preferred LMMAs to MPAs (95%). Respondents commented in open‐ended responses that they preferred LMMAs because LMMAs were local and protected their resources; also, some respondents reported that they felt informed, a sense of ownership, and control over rules. The nearby MPA was viewed negatively. Respondents claimed it was not helping local people and expressed mistrust of the MPA.

## DISCUSSION

We measured satisfaction with LMMA outcomes among adopters in northeast Madagascar using established satisfaction research methods from consumer research, IPA (Martilla & James, 1977) and NPS (Reichheld, 2003). Most outcomes needed improving to increase satisfaction among adopters, including the impacts on traditions, conflict, and connections and the impacts of the funding and livestock provided by the NGO partner. These are not necessarily independent because the cause of conflict was linked to the distribution of benefits and weakening of traditions and customs. Although the respondents expressed discontent with the LMMA, they also expressed the wish to keep it. This research can be used to increase satisfaction with and likely spread of LMMAs and other conservation initiatives and help identify negative impacts of conservation on communities for ethical conduct (CBD, 2011).

High levels of satisfaction were reported in the IPA for 2 LMMA attributes: restrictions on fishing and control over resources. Positive perceptions of fishery restrictions were surprising because fisherfolk are considered resistant to fishery restrictions in MPAs and no‐take zones (Sanchirico et al., 2006; Suuronen et al., 2010). However, looking at the results, restrictions on fishing clustered with control over the resource in the component self‐determination. Increased levels of control and participation in setting the rules in LMMAs are reported elsewhere as reasons LMMAs are preferred to MPAs in some cases (Cinner & Huchery, 2014). Local customary rules, dinas, may be more reliable in Madagascar than top‐down rules due to political instability (Newell et al., 2019). In contrast to local customary rule, MPAs can weaken local governance and tenure (Sowman & Sunde, 2018). Consistent with this, control over their resource and villages setting their own LMMA rules were cited by respondents as reasons for preferring LMMAs to the nearby MPA. Respondents reported the MPA was not helping local people, whereas the LMMA was protecting their resources. These positive perceptions were common to leaders and nonleaders, a phenomenon observed elsewhere (Long, 2017; Oliver et al., 2015) that provides a strong basis for the spread of LMMAs through positive peer referrals (Dearing & Cox, 2018; Rogers, 2003).

Despite high levels of engagement in the decision to adopt LMMAs and the wish to keep the LMMA among respondents, the IPA plot indicated only 2 attributes were perceived to have performed well, and the NPS was negative. Negative perceptions of adopting innovations are particularly detrimental to their spread (Ramirez et al., 2014). The IPA result indicating dissatisfaction with most of the LMMA attributes tested is not unprecedented (Agyare et al., 2015). By comparison, studies in tourism show that 40–100% of IPA return positive results (Azzopardi & Nash, 2013). Comparing the negative NPS to other conservation studies is not possible due to a lack of similar research. However, negative scores in the industry suggest adopter dissatisfaction, which may hinder innovation spread (Blasberg et al., 2008; Morgan et al., 2004).

Although the IPA and NPS approaches returned negative results, calculating average performance ratings returns a positive result. This difference may be because IPA sets thresholds that must be passed to achieve good work status (Lai & Hitchcock, 2015) and NPS discards somewhat better scores (Blasberg et al., 2008), and somewhat better comprised the majority of responses in our study. Additionally, NPS subtracts detractors from promoters, further reducing the final score. Although conservation evaluations typically measure changes in outcomes (e.g., material wealth), they report averages of all the data, and all scores above zero are interpreted as positive outcomes for respondents (Ahmadia et al., 2015; Gurney et al., 2014; Mascia et al., 2017). However, the word *positive* can be ambiguous unless respondents are asked their interpretation. For example, LMMA evaluations show positive social and economic impacts on participating villages in Madagascar (Barnes‐Mauthe et al., 2015; Gardner et al., 2020; Oliver et al., 2015), but they do not measure adopter satisfaction with those changes. Additionally, IPA seeks respondents’ opinions of which attributes are important to them and focuses attention on these responses (Lai & Hitchcock, 2015).

Although our analyses do not prove that IPA or NPS has accurately measured adopters’ sentiments, we consider that the use of these methods—sought from the perspectives of LMMA adopters—allows local people to be heard and their views to be acted on with minimal interpretation. Given that conservation research often involves NGOs supporting LMMAs, as in our case, using customer satisfaction methods that seek respondents’ direct priorities and opinions can help mitigate possible conflicts of interest, although fully independent research is recommended (Wahlén, 2014).

We did not find in the literature instances of researchers measuring adopters’ satisfaction with LMMAs, although peer‐to‐peer communication of satisfaction may be implied given that, in Kenya at least, initiation of new LMMAs was invariably triggered by an exchange visit to an existing LMMA (Kawaka et al., 2017). However, further research is needed before any conclusions can be drawn, and the use of customer satisfaction research methods may provide insights not gained with other methods.

Four attributes of LMMAs were identified in the IPA as important but had become worse since the LMMA was established and therefore may require improvement. This included the funding and livestock provided to villages establishing LMMAs by an NGO, which clustered in the PCA on a component we termed *NGO support*. Traditionally, increases in financial and material wealth are reported as “positive” outcomes of marine reserves (Andam et al., 2010; Ban et al., 2019; Oldekop et al., 2016). Responses that provisions of funding and livestock had made matters worse were attributed in part to inequitable distribution of benefits, known to foster conflict and undermine cooperation (Gurney et al., 2021). Conflict can also occur when NGO goals are misaligned with those of communities adopting conservation initiatives (Parker et al., 2024). Because relationships with extension officers affect satisfaction (Schilling et al., 2019) and NGOs promoting conservation initiatives are analogous to extension officers, the relationship with the NGO could affect satisfaction and initiative spread.

A negative performance rating was also found for conflict, which is not unusual for evaluations of protected areas or LMMAs (Gardner et al., 2020; Long et al., 2021; Parker et al., 2024). However, this raises ethical concerns and jeopardizes support for and spread of these conservation initiatives (Bennett & Dearden, 2014; Bennett et al., 2017). Respondents reported that conflict was more severe during the early stages of adopting and negotiating LMMA rules and eased over time. However, IPA, NPS, and mean performance ratings are limited to capturing only overall trends to one point in time and not different rates and arcs of change over time between attributes. Given the importance of conflict to respondents, further research into the causes and resolution mechanisms is recommended (Ostrom, 2009). Also, when interpreting the plot for project evaluation purposes, for example, the placement of thresholds between zones in the IPA plot can affect how attributes are prioritized for improvement (Chen, 2014). We used a scale‐centered plot because it was most relevant for this research; however, some may select target‐driven thresholds (Tonge & Moore, 2007) (e.g., do no harm and no net loss targets [Arlidge et al., 2018; Bull et al., 2018]), and the threshold of concern for conflict may be much lower than that of other attributes. Village traditions and customs were rated as having weakened since establishing LMMAs, and this strongly correlated with negative ratings for funding and livestock from the NGO. Weakening of village traditions contrasts with literature on LMMAs in Fiji, where LMMAs are considered to strengthen traditions (Aburto et al., 2015; Clarke & Jupiter, 2010; Jupiter et al., 2014; Weeks & Jupiter, 2013). The reasons for respondents feeling their traditions had become weaker were not clear, but translocation of initiatives between cultures requires locally relevant refinements and context‐specific understandings of cultural values (Infield et al., 2018). Ethnographic research in Madagascar indicates that the involvement of conservation and other external agencies in dinas, especially where full consensus is not achieved, may reconfigure Indigenous sovereignty rather than strengthen local customary traditions (Klein, 2024). Additionally, dissatisfaction was expressed significantly more by respondents of lower social standing (those who identified as nonleaders), a finding mirrored in other work (Setiawan et al., 2012), which warrants further research to ensure LMMAs are equitably managed (Leadley et al., 2014) and to avoid negative impacts that could be unethical and could constrain LMMA spread (Blasberg et al., 2008). Significant differences in opinions between genders were not found.

The prevalence of negative impacts perceived from adopting the LMMAs studied strengthens calls for future conservation initiatives to do no harm (Greiber, 2009) and could suggest the need for mandatory a priori impact assessments in line with many industries and academia (Brittain et al., 2020; Saeed et al., 2012; Wathern, 1992). Conservation initiatives meet many of the criteria used when screening for projects that require compulsory environmental impact assessments. For example, conservation initiatives are designed to affect nature (Ahmadia et al., 2015) often through purposively changing human behaviors (Dobson et al., 2019) and have the potential to significantly affect people, their livelihoods, resource use, resource rights, and culture by design and default (Lewison et al., 2019). Conservation initiatives are also often targeted toward the most vulnerable environments and endangered species (Groves et al., 2002) in the most remote regions, where impacts on nature and people may be most significant (O'Leary et al., 2018).

Broader adoption of social science methods, such as customer satisfaction approaches, in conservation science could help advance understanding of adopters’ perceptions of the outcomes of conservation and the implication this has for the spread and ethics of conservation. IPA, for example, and NPS may help identify individuals or groups with the greatest concerns and what these concerns are so that these can be prioritized for resolution with limited resources.

We conclude that measuring and improving attributes that adopters perceive as important but as perform poorly and addressing concerns of the most dissatisfied adopters may help LMMAs spread (Blasberg et al., 2008; Dearing & Cox, 2018; Martilla & James, 1977). They may also help address concerns raised about conservation initiatives having negative impacts on communities (Bennett et al., 2017). Further research could examine whether improving satisfaction among adopters of other conservation initiatives increases spread and reduces the need for inputs by conservation organizations. Additionally, comparing satisfaction among adopters of conservation initiatives that spread through peer‐to‐peer referrals without social protection measures versus those supported by NGOs or other agencies with social protections, such as funding and provision of livestock, is worthwhile. Given IPA and NPS methods are accepted standards in commerce, even with limitations (Azzopardi & Nash, 2013; Bendle et al., 2019), their use in conservation evaluations is worth further consideration. Conservation adopters may differ from customers but are similar to customers in that their perceptions of adopting initiatives and their communications may affect the adoption choices of others. Therefore, we consider customer satisfaction and NPS methods appropriate. Further research to examine whether the results from these methods correlate with referrals leading to new adoptions would be useful. Our study and approach can also be used to inform future preadoption impact assessments to minimize and mitigate negative impacts on metrics that matter most to adopters and help conservation perform more positively for people and the planet.

## AUTHOR CONTRIBUTIONS

*Literature review, development of attributes, focus group protocol, and key informant interview instrument*: Emily Lewis‐Brown. *Survey instrument*: Emily Lewis‐Brown, Morena Mills, and Katrina Davis. *Fieldwork management*: Hope Beatty, Ando Rabearisoa, and Jeannot Ramiaramanana. *Data entry*: Hope Beatty and Emily Lewis‐Brown. *Data cleaning and analyses*: Emily Lewis‐Brown. *Revisions*: All authors.

## Supporting information



Supplementary Materials.

Supplementary Materials.

Supplementary Materials.
