# Supplementary material for: Improvements for better scaling of locally managed marine areas
Source: Conserv Biol. 2025 Jun 19;39(5):e70091. doi: 10.1111/cobi.70091 (PMC12451514; doi:10.1111/cobi.70091)
Supplement: Supplementary file 1 — Supplementary Materials. [file COBI-39-e70091-s001.pdf]

Instructions for the Interviewer

Please note; all words that are unlined are guidance notes to the interviewer.

Only the words in *italics* should be read out loud to the interviewee.

Please have the questions Q1 and Q3 to Q8 filled out before the interview starts.

Section 1. Identification

1. Unique consecutive identification number of this survey____________________________

2. Background Information sheet read out and consent given Yes / No

3. Name of interviewer ____

4. Name of translator ____

5. Name of LMMA or fishing ground____

6. Village name ______________________________ Country Code: 450 (Madagascar)

7. Interviewee Code (village name / interviewee number) ____________________________

8. Date _____________ 9. Sex ____________ 10. Start time _______________

Section 2. LMMA Characteristics

11. *What is the first thing that comes to mind when you hear "Locally Managed Marine Areas"?*

12. *On the scale below from very easy to very difficult, how easy or difficult is it to know what an LMMA is?*

Very easy	*Moderately easy*	*Neither easy nor difficult*	*Moderately difficult*	*Very difficult*

Not sure ☐

For the purposes of this survey, we can consider that Locally Managed Marine Areas (LMMAs) are areas of ocean managed by coastal communities to help protect fisheries and

In this part of the survey, I'd like you to think back to when your village was first considering whether or not to establish an LMMA. I will read out four statements about the LMMA, and I want you first to tell me which one of these four statements your village thought would be the BEST thing about establishing an LMMA, and then tell me which one of these four statements the village thought would be the WORST thing about an LMMA. This is repeated several times with different options available.

13. *When your village was deciding whether or not to have an LMMA, which one of these four statements was thought by your village to be the BEST thing about an LMMA? And which one was thought by your village to be the WORST thing about an LMMA?*

8 CHOICE SETS with 4 attributes each listed according to a balanced block design

**BEST**		**WORST**

Best-Worst Scaling Choice Experiment Attribute Statements.

Item number	Choice Item Statement
1	The amount of money we could earn
2	The incentives for villages to establish and run an LMMA
3	Possible connections to other people beyond the village
4	The control we would have over our marine resources
5	New habits that might be needed
6	Whether LMMAs can be tested or changed to meet our needs
7	How complicated or simple it seemed to establish an LMMA
8	Someone in the village had seen or heard about an LMMA and its impacts
9	The benefit to future generations
10	The restriction of certain activities in an LMMA
11	The level of conflict in or beyond the village
12	The health of the environment (non-target species)
13	The monitoring and enforcement of the rules
14	The food we could catch to eat
15	The funding and training provided by an NGO
16	The pressure on the resources due to more people fishing

Bloc design for attributes in each of the 8 choice sets. Each survey had eight choice sets, each with four attributes selected according to the table below. There are eight versions of the bloc design to balance co-appearance. Each attribute appears twice per survey.

Version	Set	Attribute1	Attribute2	Attribute3	Attribute4
1	1	14	7	3	13
1	2	13	4	11	2
1	3	4	10	8	9
1	4	11	12	5	1
1	5	7	16	10	12
1	6	9	3	1	15
1	7	5	14	6	8
1	8	6	2	15	16
2	1	16	9	14	11
2	2	8	13	16	1
2	3	15	12	4	14
2	4	10	15	13	5
2	5	5	9	7	2
2	6	1	6	7	4
2	7	3	6	11	10
2	8	2	8	12	3
3	1	7	14	9	6
3	2	11	5	13	3
3	3	12	15	2	16
3	4	8	11	15	7
3	5	13	9	12	6
3	6	14	1	2	10
3	7	16	5	3	4
3	8	10	4	8	1
4	1	9	2	8	13
4	2	2	4	5	14
4	3	15	8	3	7
4	4	4	11	6	15
4	5	1	6	13	12
4	6	10	16	9	11
4	7	3	1	14	16
4	8	7	10	12	5
5	1	5	8	1	9
5	2	1	7	2	11
5	3	6	5	16	8
5	4	12	11	14	15
5	5	9	3	4	12
5	6	4	13	16	7
5	7	6	3	10	2
5	8	14	13	10	15
6	1	12	2	9	3
6	2	11	1	6	4

6	3	15	5	1	9
6	4	13	15	10	8
6	5	8	14	11	12
6	6	16	10	4	3
6	7	2	7	13	6
6	8	16	7	5	14
7	1	4	13	12	5
7	2	11	14	9	13
7	3	6	12	7	10
7	4	3	11	7	8
7	5	5	3	15	1
7	6	14	1	2	10
7	7	9	16	6	15
7	8	2	16	4	8
8	1	6	4	14	5
8	2	13	3	16	11
8	3	12	13	1	16
8	4	15	2	1	7
8	5	8	12	10	9
8	6	10	11	5	2
8	7	3	8	14	6
8	8	7	15	9	4

14. *Was anything else important to your village when deciding whether or not to establish an LMMA, which we have not discussed yet?*

<u>If the interviewee gives one word answers, such as “education”, “tourism”, or “goats” please ask why this was important or what was that was important.</u>

Thank you, that was very helpful.

15. *How easy or difficult was it to think back to when your village was first deciding whether or not to establish an LMMA?*

Very easy	*Somewhat easy*	*Neither easy nor difficult*	*Somewhat difficult*	*Very difficult*

Not sure ☐

16. *How easy or difficult was it to answer these questions on behalf of the village?*

Very easy	*Somewhat easy*	*Neither easy nor difficult*	*Somewhat difficult*	*Very difficult*

Not sure ☐

Section 3. Decision-making

17a. *Does your village currently have an LMMA?* YES / NO

☐ If YES

b. *Does your village want to keep it*? Yes / No

c. *Please EXPLAIN why*

18. *How much did you agree or disagree with the decision about whether or not to establish an LMMA?*

Strongly Agreed	*Slightly Agreed*	*Neither agreed nor Disagreed*	*Slightly Disagreed*	*Strongly Disagreed*

Not sure ☐

18b. *If you strongly agreed or strongly disagreed, why?*

Not sure ☐

Thank you. Now, please can you tell me a little more about LMMAs

19. *On the scale below from very easy to very difficult, how easy is it to know where the boundaries are?*

Very easy	*Moderately easy*	*Neither easy nor difficult*	*Moderately difficult*	*Very difficult*

☐

20. *On the scale below from very easy to very difficult, how easy is it to know what the rules are?*

Very easy	*Moderately easy*	*Neither easy nor difficult*	*Moderately difficult*	*Very difficult*

Not sure ☐

Great, thanks. Next I would like to understand how the decision about whether or not to establish an LMMA was made.

21. *How would you describe your role when the decision of whether or not to establish an LMMA was made?*

☐ Village representative, Council member, Vice President, or President
☐ Other (*please explain below*)

22. *How involved were you in the decision of whether or not to establish an LMMA?*

I was unaware of the decision				***I was very involved in the decision***
1	2	3	4	5

Not sure ☐

Thank you very much

For the next question, I don't need names or details, but a Yes / No answer would be good:

23a. *Was there a particular individual who was influential in the decision about whether or not to establish an LMMA?* Yes / No / Not sure
23b. *Someone within the village*: Yes / No / Not sure
23c. *Someone from outside the village*: Yes / No / Not sure

Section 4. Perceived outcomes

Now we would like to know whether things have changed since the village decided whether or not to establish an LMMA. I will read out a sentence and I would like you to tell me if things have changed between then and now, on a scale from: Much Better; Moderately better; Neither better nor worse; Moderately worse; Much worse; or Not sure.

24. *Have incomes in the village changed?*

Much better	*Moderately better*	*Neither better nor worse*	*Moderately worse*	*Much worse*

Not sure ☐

25. *Has the amount of food the village can catch to eat changed?*

Much better	*Moderately better*	*Neither better nor worse*	*Moderately worse*	*Much worse*

Not sure ☐

26. *Have the connections to other people in the village and beyond the village changed?*

Much better	*Moderately better*	*Neither better nor worse*	*Moderately worse*	*Much worse*

Not sure ☐

27. *Does the village have more or less control over marine resources?*

Much more	*A little more*	*Neither more nor less*	*A little less*	*Much less*

Not sure ☐

28. *Has the level of conflict changed?*

Much better	*Moderately better*	*Neither better nor worse*	*Moderately worse*	*Much worse*

Not sure ☐

29. *Has the health of the environment changed? (non-target species)*

Much better	*Moderately better*	*Neither better nor worse*	*Moderately worse*	*Much worse*

Not sure

30. *Has the pressure on resources changed?*

Much better	*Moderately better*	*Neither better nor worse*	*Moderately worse*	*Much worse*

Not sure

Next, I'd like to ask you a little more about the changes in the sea. Comparing your village now to the year your village decided whether or not to establish an LMMA, has your village noticed any differences in the following things?

31. *Has there been a change in the types of fish and other animals in the sea?*

Greatly increased	*Moderately increased*	*Neither increased nor decreased*	*Moderately decreased*	*Greatly decreased*

Not sure

32. *Has the condition of the habitats changed (e.g. corals, mangroves, sea grasses)*

Much better	*Moderately better*	*Neither better nor worse*	*Moderately worse*	*Much worse*

Not sure

33. *Has the overall quality of life changed for the village*

Much better	*Moderately better*	*Neither better nor worse*	*Moderately worse*	*Much worse*

Not sure

Much stronger	*Moderately stronger*	*Neither stronger nor weaker*	*Moderately weaker*	*Much weaker*

Not sure ☐

35. *Do you think the distribution of positive and negative impacts from the management here are more fair or less fair than before?*

Much more fair	*Moderately more fair*	*Neither more fair nor less fair*	*Moderately less fair*	*Much less fair*

Not sure ☐

36. *Has tourism changed in the area?*

Much better	*Moderately better*	*Neither better nor worse*	*Moderately worse*	*Much worse*

Not sure ☐

37. *Have people's understanding of the environment changed?*

Much better	*Moderately better*	*Neither better nor worse*	*Moderately worse*	*Much worse*

Not sure ☐

Thank you. Now, I would like to ask how things are working for your village since the village made the decision about whether or not to establish an LMMA here.

38. *If your village restricted any fishing activities or gears, has this been good or bad?*

Very good	*Moderately good*	*Neither good nor bad*	*Moderately bad*	*Very bad*

Not sure ☐

39. *If your village received any chickens, goats etc, did they make life different?*

Much better	*Moderately better*	*Neither better nor worse*	*Moderately worse*	*Much worse*

Not sure ☐

40. *If your village received any funding or training, was it helpful or not?*

Very helpful	*Moderately helpful*	*Neither helpful nor unhelpful*	*Moderately unhelpful*	*Very unhelpful*

Not sure ☐

41. *If people have needed to establish new habits, has this been difficult or easy?*

Very easy	*Moderately easy*	*Neither easy nor difficult*	*Moderately difficult*	*Very difficult*

Not sure ☐

Thank you, that was really useful.

42a. *Did you hear about a proposal for an MPA in the area?* Y / N / not sure

42b. *What does the village think of MPAs in comparison to LMMAs and Why?*

Lastly, remembering this is confidential, I'd like to ask you a few questions about yourself.

43a. *What are your main sources of income;*
43b. What proportion are they of your total income?
If they say 'fishing' please ask them to be more specific – what they catch, how and where.

	less than half	*about half*	*more than half*	Not sure
i				
ii				
iii				

iv				

44. *What proportion of your household's income is dependent on the sea?*

None	*Less than half*	*Around half*	*More than half*	*All*

☐ Don't know

45. *Please can you tell me your approximate income weekly in Malagasy Ariary?*

☐ *0 to 50,000* ☐ *50,001 to 100,000* ☐ *100,001 to 200,000*

☐ *above 200,000* ☐ don't know

46. *How many community organisations are you involved in?* _____________

47. *What is your age?* (please tick one category)

18-25 _____ *26-45* _____ *46-65* _____ *66+* _____ Not sure _____

48. *How many years did you spend at school?* __________ Not sure _____

49. *Can you read and write?* Yes / No / Not sure

Many thanks. And finally,

50. *Do you have any comments about this survey or about locally managed marine areas that we have not discussed yet?*

THANK YOU VERY MUCH FOR YOUR TIME. RECORD End time:______________
