# Supplementary material for: Improvements for better scaling of locally managed marine areas
Source: Conserv Biol. 2025 Jun 19;39(5):e70091. doi: 10.1111/cobi.70091 (PMC12451514; doi:10.1111/cobi.70091)
Supplement: Supplementary file 2 — Supplementary Materials. [file COBI-39-e70091-s003.docx]

# Supplementary Information 2

**S2.0 Importance-Performance Analysis method (**[**Martilla & James, 1977**](#_ENREF_7)**).** The simplest form of this analysis can be completed as follows: identify the most useful attributes to study e.g. through a literature search, key informant interviews, focus groups. Design survey questions measuring respondents’ perceptions of the importance and performance of each attribute, e.g. using matrix questions. Use equal intervals in the response options. Construct a database with one row per respondent. Calculate means of the importance and the performance of each attribute and plot an Importance-Performance Analysis matrix with the most relevant quadrant thresholds for your study. Consider including a diagonal division on the plot ([Azzopardi & Nash, 2013](#_ENREF_2)). Extensions of this Importance-Performance Analysis method and statistical analyses are possible ([Matzler, Sauerwein, & Heischmidt, 2003](#_ENREF_8); [Abalo, Varela, & Manzano, 2007](#_ENREF_1); [Wu & Shieh, 2009](#_ENREF_11); [Azzopardi & Nash, 2013](#_ENREF_2); [Chen, 2014](#_ENREF_3); [Feng, Mangan, Wong, Xu, & Lalwani, 2014](#_ENREF_4); [Lai & Hitchcock, 2015b](#_ENREF_6), [2015a](#_ENREF_5); [Sever, 2015](#_ENREF_10); [Ormanovi, 2017](#_ENREF_9)).

| Please rate the importance of each attribute of the food you ate | | | | | |  |  |
| --- | --- | --- | --- | --- | --- | --- | --- |
| IMPORTANCE | Not at all important |  |  |  | Extremely important | Don't know | Not applicable |
|  | 1 | 2 | 3 | 4 | 5 |  |  |
| Colour |  |  |  |  |  |  |  |
| Eco-friendly |  |  |  |  |  |  |  |
| Healthy |  |  |  |  |  |  |  |
| Packaging |  |  |  |  |  |  |  |
| Portion size |  |  |  |  |  |  |  |
| Price |  |  |  |  |  |  |  |
| Smell |  |  |  |  |  |  |  |
| Taste |  |  |  |  |  |  |  |
| Temperature |  |  |  |  |  |  |  |
| Texture |  |  |  |  |  |  |  |

| Please rate the performance of each attribute of the food you ate | | | | | |  |  |
| --- | --- | --- | --- | --- | --- | --- | --- |
| PERFORMANCE | Extremely poor performance |  |  |  | Extremely good performance | Don't know | Not applicable |
|  | 1 | 2 | 3 | 4 | 5 |  |  |
| Colour |  |  |  |  |  |  |  |
| Eco-friendly |  |  |  |  |  |  |  |
| Healthy |  |  |  |  |  |  |  |
| Packaging |  |  |  |  |  |  |  |
| Portion size |  |  |  |  |  |  |  |
| Price |  |  |  |  |  |  |  |
| Smell |  |  |  |  |  |  |  |
| Taste |  |  |  |  |  |  |  |
| Temperature |  |  |  |  |  |  |  |
| Texture |  |  |  |  |  |  |  |

**Figure S2.1a**     Perceived performance (a. mean, b. median and c. mode) of the ten attributes used in the Importance-Performance Analysis (n88).

**
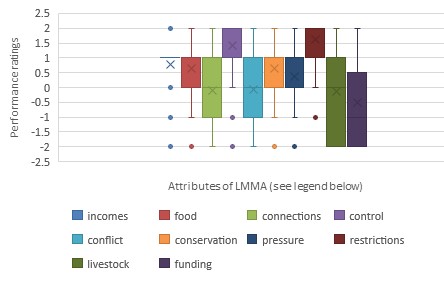
**

**Figure S2.1b** Distribution of perceived performance data of the ten attributes used in the Importance-Performance Analysis (n88).


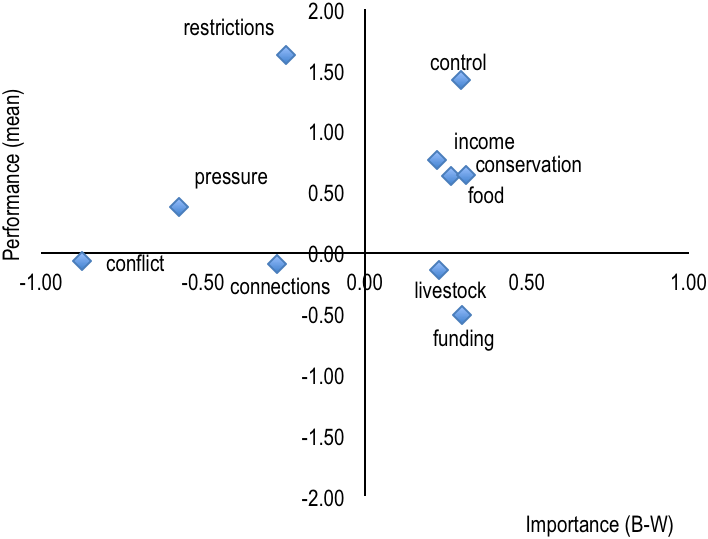


**S2.2** Importance-Performance scale-centred matrix plot with best minus worst to rank attribute importance. Importance (x-axis) indicates the relative importance of attributes derived from a best-worst scaling choice experiment (best minus worst) where -1 would indicate an attribute ranked as the worst attribute of LMMAs of those presented in the choice experiment 100% of the time is occurred in the choice experiment (100%), and +1 would indicate an attribute ranked as the best attribute of LMMAs 100% of the times it occurred in the choice experiment. In this plot, -1 and +1 both indicate highly important attributes. Performance (y-axis) indicates perceived performance of attributes rated on scale based questions, where respondents rated attributes as having become much worse (-2), through neither better nor worse (0), to much better (+2) over the course of the decade of having had the LMMA. Thus, the attribute relating to funding for the LMMA was rated as somewhat important but this attribute was perceived by respondents on average to have performed somewhat negatively. Meanwhile, restrictions on fishing was ranked as a slightly negative attribute but was perceived to have performed well for respondents on average. Green shading in the top right and top left of the IPA plot indicates zones where attributes falling are important and performing well. Any attribute falling below the two diagonal lines require attention, and attributes in the bottom right and left corners of the plot (shaded red) would be indicated for primary focus for improvement to increase customer satisfaction.

**S2.3**  Perceived performance (frequency counts) 18 attributes of LMMAs on a scale from “much worse” to “much better” (n = 88)

**S2.4** Perceived performance of LMMAs on 17 attributes (mean) for leaders (n = 46) and non-leaders (n = 42) on a scale of “much worse” (-2) through “neither worse nor better” (0) to “much better” (+2). Sifferences between the responses of leaders and non-leaders were non-significant, other than funding and traditions which returned significant differences in responses (Fisher’s Exact test, *p* = 0.002, and *p* = 0.008 respectively). * denotes attributes with significant differences between leaders and non-leaders.

(a)


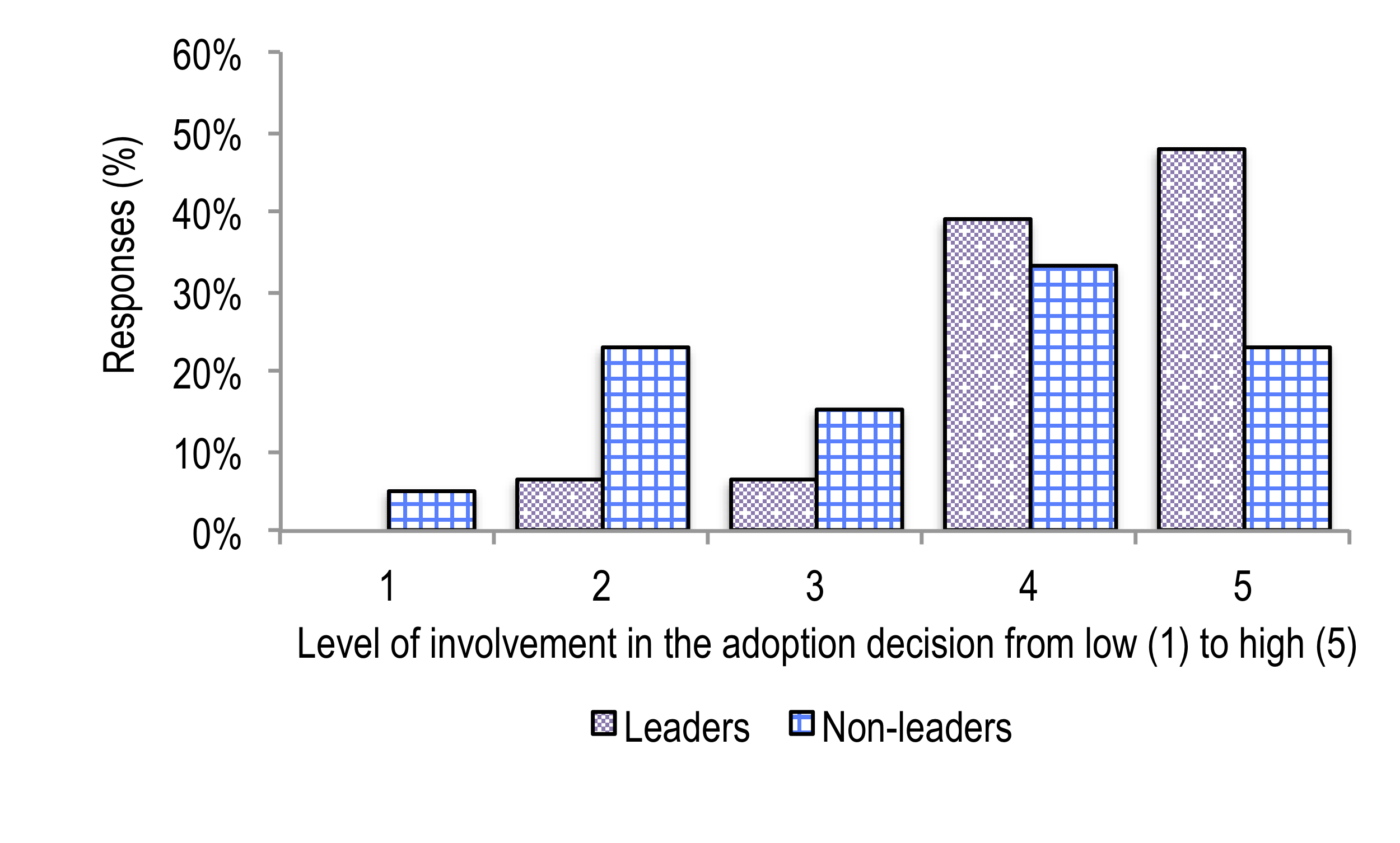


(b)


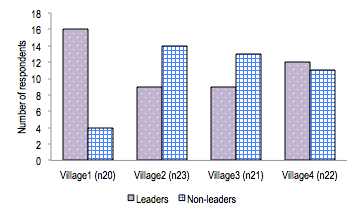


(c)


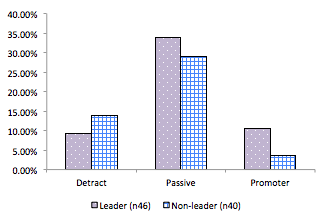


**S2.5** The distribution of respondents who self-reported as leaders and non-leaders. (a) indicates the level of involvement leaders and non-leaders reported as having had in the decision of whether to adopt an LMMA which is significantly different between these groups (Mann Whitney U z-score 3.05, *p* = <0.01, n = 88), (b) who responded from the four villages surveyed (c) who were detracters, passive or promoters in the NPS (percentage).

(a)

(b)

**S2.6** Perceived impact on quality of life following establishment of a Locally Managed Marine Areas (LMMAs) on a 5-point Likert-style scale for villages 1 to 4. The divergent bar plot (a) shows the percentage of responses in 5 categories, from left to right: ‘Much worse’ (dark red narrow horizonal hatching ~ none shown as none recorded); ‘Somewhat worse’ (pale red wide horizontal hatching); ‘Neither better nor worse’ (solid grey shading); ‘Somewhat better’ (pale green wide diagonal hatching); to ‘Much better’ (dark green narrow diaganol hatching) with mean scores per village shown. While (b) shows the Net Promoter Score calculated on the same question and data for each village, where NPS per village is shown, and calculated as: promoters (responses of ‘Much better’) minus detracters (responses of ‘Neither better nor worse’, ‘Moderately worse’ or ‘Much worse’) (n = 88).

**
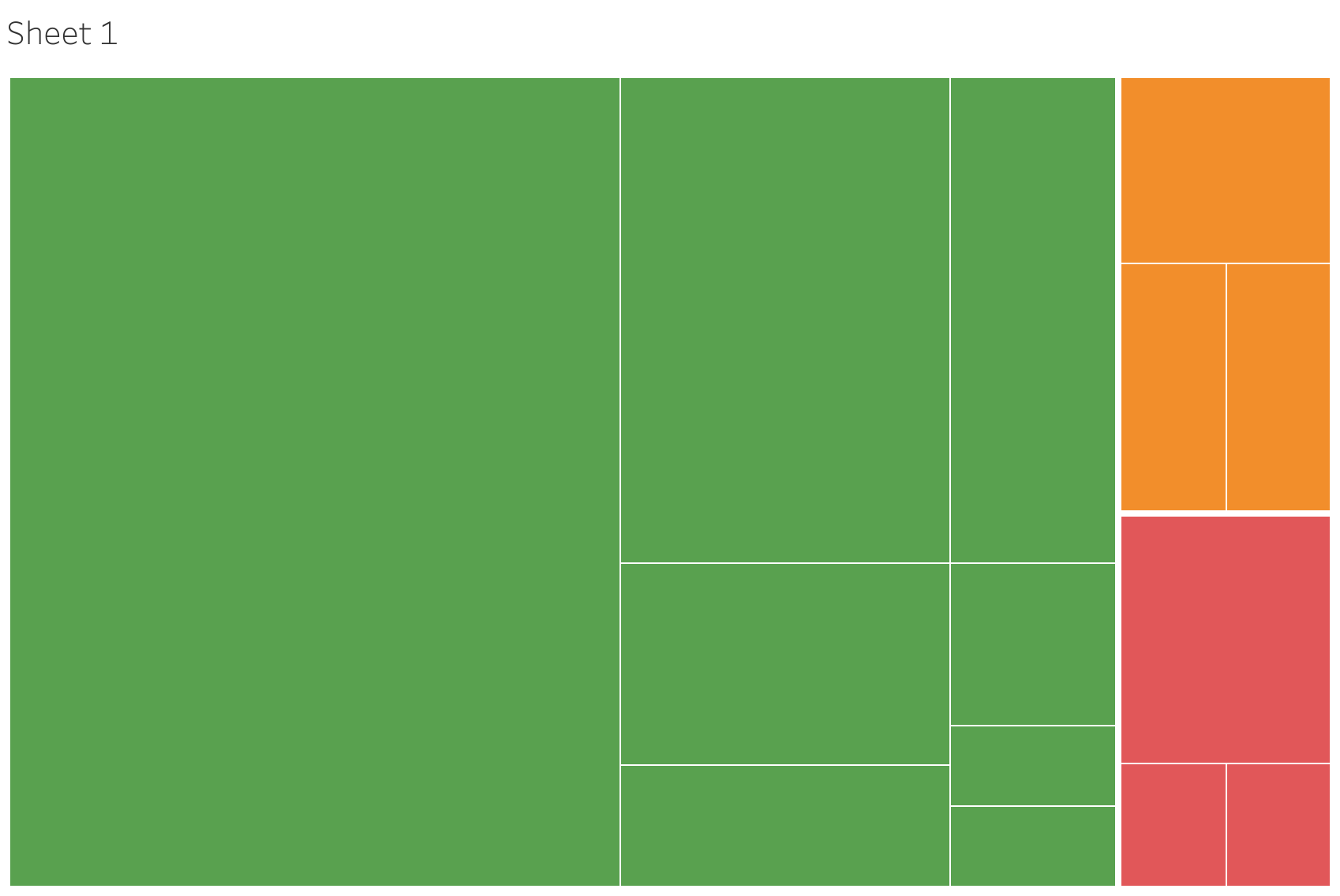
**

Unfair distribution of livestock

Rules are helpful

Diffusion desirable

Benefits

from the LMMA

Good fishery association

Conditional support

Improvements needed

Requests made

Future generations

Unsatisfied

Conflict

Advice is good

Keep

Resources

are protected and/or improving due to the LMMA

**S2.7** Relative frequency of reasons shared in an open box question which asked why respondents might wish to either keep or abandon the LMMAs studied (n80). Colours represent positive (green), mixed (amber), or negative (red) reponses to the question of whether respondents would like to keep their LMMA and why.

_

_

_

_

_

_

_

_

_

**S2.8a** Principle Component Analysis of Locally Managed Marine Areas (LMMAs) performance ratings by respondents on scale-based questions from “much better (+2) to “much worse (-2) for ten attributes (n = 86). C1 (comprised of livestock and funding) explained 28.84% of variance and C2 (comprised of pressure, conservation and food) explained 20.66% of variance. Two further components were discovered: C3 (comprised of control, restrictions and incomes) explained 14.79% of variance, and C4 (comprised of conflict and connections) explained 11.61% of variance.

**S2.8b** Principle Component Analysis Pattern Matrix (the correlations between each attribute and each component). S1a shows the influence of each factor on each attribute, and S1b shows the relative positions of each attribute of each factor. Colour indicates the coefficients that cluster to form a component with high (yellow), medium (grey) and weak correlations (white).

|  | **C1 natural resources** | **C2 NGO support** | **C3 self-determination** | **C4 relationships** |
| --- | --- | --- | --- | --- |
| **Conservation** | 0.871 | -0.201 | 0.104 | 0.068 |
| **Pressure** | 0.741 | 0.319 | -0.030 | -0.215 |
| **Food** | 0.489 | -0.202 | -0.461 | 0.269 |
| **Funding** | 0.007 | -0.952 | 0.005 | -0.127 |
| **Livestock** | 0.014 | -0.910 | -0.130 | -0.024 |
| **Control** | -0.311 | -0.065 | -0.828 | -0.107 |
| **Restrictions** | 0.097 | 0.057 | -0.798 | -0.038 |
| **Income** | 0.320 | -0.125 | -0.638 | 0.280 |
| **Conflict** | -0.015 | 0.314 | -0.076 | 0.866 |
| **Connections** | -0.069 | -0.430 | 0.214 | 0.679 |

Extraction Method: Principal Component Analysis. Rotation Method: Oblimin with Kaiser Normalization.

**S2.8c**

| **C1 natural resources** | | **C2 NGO support** | | **C3 self-determination** | | **C4 relationships** | |
| --- | --- | --- | --- | --- | --- | --- | --- |
| **Conservation** | 0.871 | Pressure | 0.319 | Connections | 0.214 | Conflict | 0.866 |
| **Pressure** | 0.741 | Conflict | 0.314 | Conservation | 0.104 | Connections | 0.679 |
| **Food** | 0.489 | Restrictions | 0.057 | Funding | 0.005 | Income | 0.280 |
| Income | 0.320 | Control | -0.065 | Pressure | -0.030 | Food | 0.269 |
| Restrictions | 0.097 | Income | -0.125 | Conflict | -0.076 | Conservation | 0.068 |
| Livestock | 0.014 | Conservation | -0.201 | Livestock | -0.130 | Livestock | -0.024 |
| Funding | 0.007 | Food | -0.202 | Food | -0.461 | Restrictions | -0.038 |
| Conflict | -0.015 | Connections | -0.430 | Income | -0.638 | Control | -0.107 |
| Connections | -0.069 | Livestock | -0.910 | Restrictions | -0.798 | Funding | -0.127 |
| Control | -0.311 | Funding | -0.952 | Control | -0.828 | Pressure | -0.215 |
| Extraction Method: Principal Component Analysis. | | | |  |  |  |  |
| Rotation Method: Oblimin with Kaiser Normalization. | | | |  |  |  |  |

**S2.8d** Principle Component Analysis Total Variance Explained

| Component | Initial Eigenvalues |  |  | Extraction Sums of Squared Loadings |  |  | Rotation Sums of Squared Loadings |
| --- | --- | --- | --- | --- | --- | --- | --- |
|  | **Total** | **% of Variance** | **Cumulative %** | **Total** | **% of Variance** | **Cumulative %** | **Total** |
| 1 | 2.884 | 28.844 | 28.844 | 2.884 | 28.844 | 28.844 | 1.948 |
| 2 | 2.066 | 20.658 | 49.502 | 2.066 | 20.658 | 49.502 | 2.317 |
| 3 | 1.479 | 14.789 | 64.291 | 1.479 | 14.789 | 64.291 | 2.205 |
| 4 | 1.161 | 11.61 | 75.902 | 1.161 | 11.61 | 75.902 | 1.6 |
| 5 | 0.757 | 7.57 | 83.472 |  |  |  |  |
| 6 | 0.535 | 5.353 | 88.825 |  |  |  |  |
| 7 | 0.442 | 4.419 | 93.244 |  |  |  |  |
| 8 | 0.357 | 3.569 | 96.814 |  |  |  |  |
| 9 | 0.181 | 1.814 | 98.628 |  |  |  |  |
| 10 | 0.137 | 1.372 | 100 |  |  |  |  |

**REFERENCES**

Uncategorized References

Abalo, J., Varela, J., & Manzano, V. (2007). Importance values for Importance–Performance Analysis: A formula for spreading out values derived from preference rankings. *Journal of Business Research, 60*(2), 115-121. doi:10.1016/j.jbusres.2006.10.009

Azzopardi, E., & Nash, R. (2013). A critical evaluation of importance–performance analysis. *Tourism Management, 35*, 222-233. doi:10.1016/j.tourman.2012.07.007

Chen, K.-Y. (2014). Improving importance-performance analysis: The role of the zone of tolerance and competitor performance. The case of Taiwan's hot spring hotels. *Tourism Management, 40*, 260-272. doi:10.1016/j.tourman.2013.06.009

Feng, M., Mangan, J., Wong, C., Xu, M., & Lalwani, C. (2014). Investigating the different approaches to importance–performance analysis. *The Service Industries Journal, 34*(12), 1021-1041. doi:10.1080/02642069.2014.915949

Lai, I. K. W., & Hitchcock, M. (2015a). A consideration of normality in importance–performance analysis. *Current Issues in Tourism, 18*(10), 979-1000. doi:10.1080/13683500.2014.990423

Lai, I. K. W., & Hitchcock, M. (2015b). Importance–performance analysis in tourism: A framework for researchers. *Tourism Management, 48*, 242-267. doi:10.1016/j.tourman.2014.11.008

Martilla, J. A., & James, J. C. (1977). Importance-Performance Analysis. *Journal of Marketing, 41*(1), 77-79.

Matzler, K., Sauerwein, E., & Heischmidt, K. (2003). Importance-performance analysis revisited: the role of the factor structure of customer satisfaction. *The Service Industries Journal, 23*(2), 112-129. doi:10.1080/02642060412331300912

Ormanovi. (2017). Importance-Performance Analysis: Different Approaches. *Acta Kinesiologica, 11* 58-66.

Sever, I. (2015). Importance-performance analysis: A valid management tool? *Tourism Management, 48*, 43-53. doi:10.1016/j.tourman.2014.10.022

Wu, H.-H., & Shieh, J.-I. (2009). Quantifying uncertainty in applying importance-performance analysis. *Quality & Quantity, 44*(5), 997-1003. doi:10.1007/s11135-009-9245-8
